# Altered profile of floral volatiles and lignin content by down-regulation of *Caffeoyl Shikimate Esterase* in Petunia

**DOI:** 10.1186/s12870-023-04203-0

**Published:** 2023-04-22

**Authors:** Joo Young Kim, Keun Ho Cho, Shea A. Keene, Thomas A. Colquhoun

**Affiliations:** 1 Environmental Horticulture Department, Plant Innovation Center, Institute of Food and Agricultural Sciences, University of Florida, 1529 Fifield Hall, Gainesville, FL 32611 USA; 2 Horticultural Sciences Department, Institute of Food and Agricultural Sciences, University of Florida, Gainesville, FL 32611 USA

**Keywords:** *Caffeoyl shikimate esterase*, *CSE*, Phenylpropanoids, Petunia, Volatiles, Lignin

## Abstract

**Background:**

The floral volatile profile of *Petunia* x *hybrida* ‘Mitchell diploid’ (MD) is dominated by phenylpropanoids, many of which are derived from *p*-coumaric acid. However, the downstream processes involved in the production of caffeoyl-CoA and feruloyl-CoA from *p*-coumaric acid are complex, as the genes and biosynthesis steps are associated with flavonoids and lignin synthesis as well as floral volatiles benzenoid/phenylpropanoid (FVBP). *Caffeoyl shikimate esterase* (*CSE*) converts caffeoyl shikimate to caffeic acid and is considered one of the essential regulators in lignin production. Moreover, CSE in involved in phenylpropanoid production. To investigate the roles of CSE in FVBP biosynthesis, we used RNAi-mediated *CSE* down-regulated (*ir-PhCSE*) petunias.

**Results:**

Lowered *CSE* transcript accumulation in *ir-PhCSE* plants resulted in reduced lignin layers in the stems and stunted growth, suggesting a positive correlation between lignin layers and lignin content. The altered *CSE* level influenced the expression of many FVBP genes, including elevated transcripts of *p-coumarate-3-hydroxylase* (*C3H*), *hydroxycinnamoyl transferase* (*HCT*), and *4-coumaric acid:* *CoA ligase* (*4CL*). In particular, the expression of *C4H* in *ir-PhCSE* plants was more than twice the expression in MD plants. Moreover, the production of volatile compounds was alterend in *ir-PhCSE* plants. Most floral volatiles decreased, and the amounts of phenylalanine and caffeic acid were significantly lower.

**Conclusions:**

Reduced lignin layers in the stems and stunted growth in *ir-PhCSE* plants suggest that PhCSE is essential for lignin production and plant growth in petunia. The decreased *CSE* level influenced the expression of many FVBP genes, and interference of shikimate derivates altered volatile compound production. Significantly decreased caffeic acid, but not ferulic acid, in *ir-PhCSE* plants suggest that CSE is primarily involved in the reaction of caffeoyl shikimate. Higher *C3H* and *C4H* transcripts seem to alleviate accumulated *p*-coumaric acid resulting from altered *CSE*. Finally, alteration in *C3H*, *HCT*, and *4CL* in *CSE* down-regulated plants suggests an interaction of the FVBP genes, leading to the regulation of floral volatiles of petunia.

**Supplementary Information:**

The online version contains supplementary material available at 10.1186/s12870-023-04203-0.

## Background

**Fig. 1:**
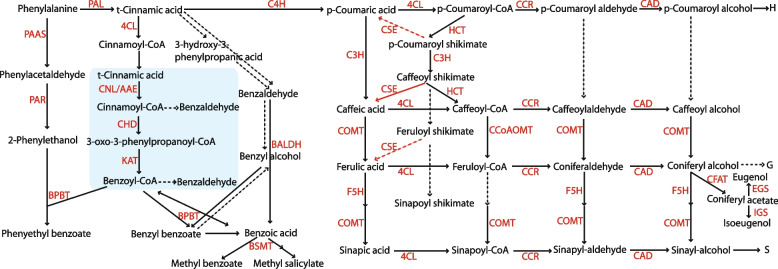
Proposed floral volatile benzenoid/phenylpropanoid biosynthesis (FVBP) pathway in *Petunia* x *hybrida* ‘Mitchell Diploid’ (MD). *BALDH*, benzaldehyde dehydrogenase; *BPBT*, benzoyl-CoA:benzylalcohol/2-phenylethanol benzoyltransferase; *BSMT*, benzoic acid/salicylic acid carboxyl methyltransferase; *4CL*, 4-coumaroyl: CoA ligase; *C3H*, *p*-coumarate 3-hydroxylase; *C4H*, cinnamate 4-hydroxylase; *CAD*, cinnamyl alcohol dehydrogenase; *CCoAOMT*, caffeoyl-CoA 3-O-methyltransferase; *CCR*, cinnamoyl-CoA reductase; *CFAT*, coniferyl alcohol acetyltransferase; *CHD*, cinnamoyl-CoA hydratase dehydrogenase; *CNL/AAE*, cinnamate:CoA ligase/acyl-activating enzyme; *COMT*, caffeic/5-hydroxyferulic acid O-methyltransferase; *CSE*, caffeoyl shikimate esterase; *EGS*, eugenol synthase; *F5H*, ferulate 5-hydroxylase; *HCT*, hydroxycinnamoyl transferase; *IGS*, isoeugenol synthase; *KAT*, 3-ketoacyl-CoA thiolase; *PAAS*, phenylacetaldehyde synthase; *PAL*, phenylalanine ammonia lyase; *PAR*, phenylalanine reductase

Floral volatiles are organic compounds with low molecular weights and high vapor pressures, which allow them to migrate through cell and plasma membranes [1, 2]. Terpenoids, benzenoids/phenylpropanoids, and fatty acid derivates are three major classes of volatiles. The benzenoids/phenylpropanoids are the second largest group of volatiles in petunia, and they are derived from the shikimic acid pathway through phenylalanine in petunia [3–5]. *Petunia* x *hybrida* ‘Michell Diploid’ (MD) is a model system for studying floral volatile benzenoids/phenylpropanoids (FVBP) because it has relatively large flowers (~ 5 cm limb diameter when fully opened) and emits significant amounts of volatiles (> 100 µg·gFW^−1^·h^−1^) [6]. The FVBP genes of petunia can be categorized into two groups: the phenylalanine ammonia lyase (PAL) group and the 4-coumaric acid: CoA ligase (4CL) group (Fig. 1). Although the functions of many genes in the PAL group have been well established [3, 7, 8], the function of many genes in the 4CL group have not been elucidated because the genes are associated with flavonoids and lignin biosynthesis, in addition to FVBP biosynthesis [9, 10]. In particular, the downstream process involved in the production of caffeoyl-CoA and feruloyl-CoA from *p*-coumaric acid is still unclear. *p*-Coumaric acid is essential to the synthesis of other phenylpropanoids which play vital roles in all aspects of plant responses, including defense mechanisms, hormones, homeostasis, stability, reproduction, and lignin production [11]. *p*-Coumaric acid is converted to *p*-coumaroyl-CoA by *4CL* and then to *p*-coumaroyl shikimate/quinate by *hydroxycinnamoyl transferase* (*HCT*). *p-Coumarate-3-hydroxylase* (*C3H*) catalyzes a reaction of *p*-coumaroyl shikimate/quinate to caffeoyl shikimate/quinate, which is subsequently converted back to caffeoyl-CoA by *HCT* [12]. Klempien et al. [13] proposed that *4CL* catalyzes the reactions of *p*-coumaric/caffeic/ferulic acid to their corresponding CoA thioesters, *p*-coumaroyl/caffeoyl/feruloyl-CoA, in petunia (Fig. 1). *p*-Coumaric/caffeic/ferulic/sinapic acids are also converted to *p*-coumaroyl/coniferyl/sinapoyl alcohols, which are the precursors for various lignin polymers [14, 15]. Lignin is a phenolic polymer derived from phenylalanine, and it is an essential structural component of plant secondary cell walls. Lignin polymers are composed of three types of units: hydroxyphenyl (H), guaiacyl (G), and syringyl (S) monomers. Their composition ratio varies depending on the plant species and organs [14]. Changes in *C3H* and *HCT* affect lignin production. Reduced lignin contents were observed in *C3H* Arabidopsis mutants [16, 17], *C3H* knock-down hybrid poplars [18], *HCT* tobacco mutants [19], and in *HCT* knock-down Arabidopsis [20].

*Caffeoyl shikimate esterase* (*CSE*) catalyzes the conversion of caffeoyl shikimate to caffeic acid [21]. Saleme et al. [22] proposed that *CSE* is associated with all reactions of *4CL* by converting *p*-coumaroyl/caffeoyl/feruloyl/sinapoyl shikimate to *p*-coumaroyl/caffeoyl/feruloyl/sinapoyl-CoA. CSE also plays an essential role in lignin biosynthesis. Down-regulation of *CSE* resulted in reduced lignin content and increased cellulose production in *CSE*-silenced Arabidopsis and poplar mutants [22, 23]. The total lignin content was 25% lower in the poplar and 65% lower in the Arabidopsis mutants. The conversion of cellulose to glucose approximately quadrupled in the Arabidopsis [21] and poplar mutants [24], and another study on poplar mutants found elevated amount of cellulose and a 62% increase in glucose production [22]. CSE is also involved in plant growth and development. In Arabidopsis, a *CSE* knockdown mutant exhibited smaller inflorescence stems [21]. A *CSE* mutant of *Medicago truncatula* with a transposon insertion resulted in severe dwarfism and altered development [25], while poplar mutants with an intron-spliced hairpin RNA of CSE were slightly decreased, though the mutants did not have significant morphological differences compared to the wild type [22]. The alteration of plant height and development in the *CSE* mutant suggests that CSE is crucial for the growth of Arabidopsis, poplar, and barrel clover (*Medicago truncatula*). However, CSE is not necessary for phenylpropanoid production in some crops, such as *Brachypodium distachyon*, corn, sorghum, and sugarcane [25, 26]. In these crops, ascorbate peroxidase converts *p*-coumaric acid to caffeic acid and produces the G and S units of lignin [27].

In a previous study, we cloned petunia *CSE* (*PhCSE*, #MF421742), and analyzed spatial and floral developmental transcript accumulation [28]. PhCSE belongs to the α/β-hydrolase superfamily, which is thought to be localized in the peripheral membrane (uniport.org, https://www.uniprot.org/uniprotkb/Q9C942/entry). *PhCSE* transcript accumulation was high in petal limb tissues and gradually increased with flower growth, and gradually decreased with flower development. In this study, we made down-regulated *CSE* petunia plants to investigate the function of CSE in the production and emission of floral volatiles. RNAi-mediated *CSE* knockdown petunias showed disturbances in plant growth, volatile profiles, and lignin synthesis. Since the roles of CSE in FVBP production have not yet been reported, this study may provide evidence for the involvement of CSE in the biosynthesis of volatiles in petunia.

## Results

### Down-regulation of *PhCSE* and characteristics of plant growth

**Fig. 2:**
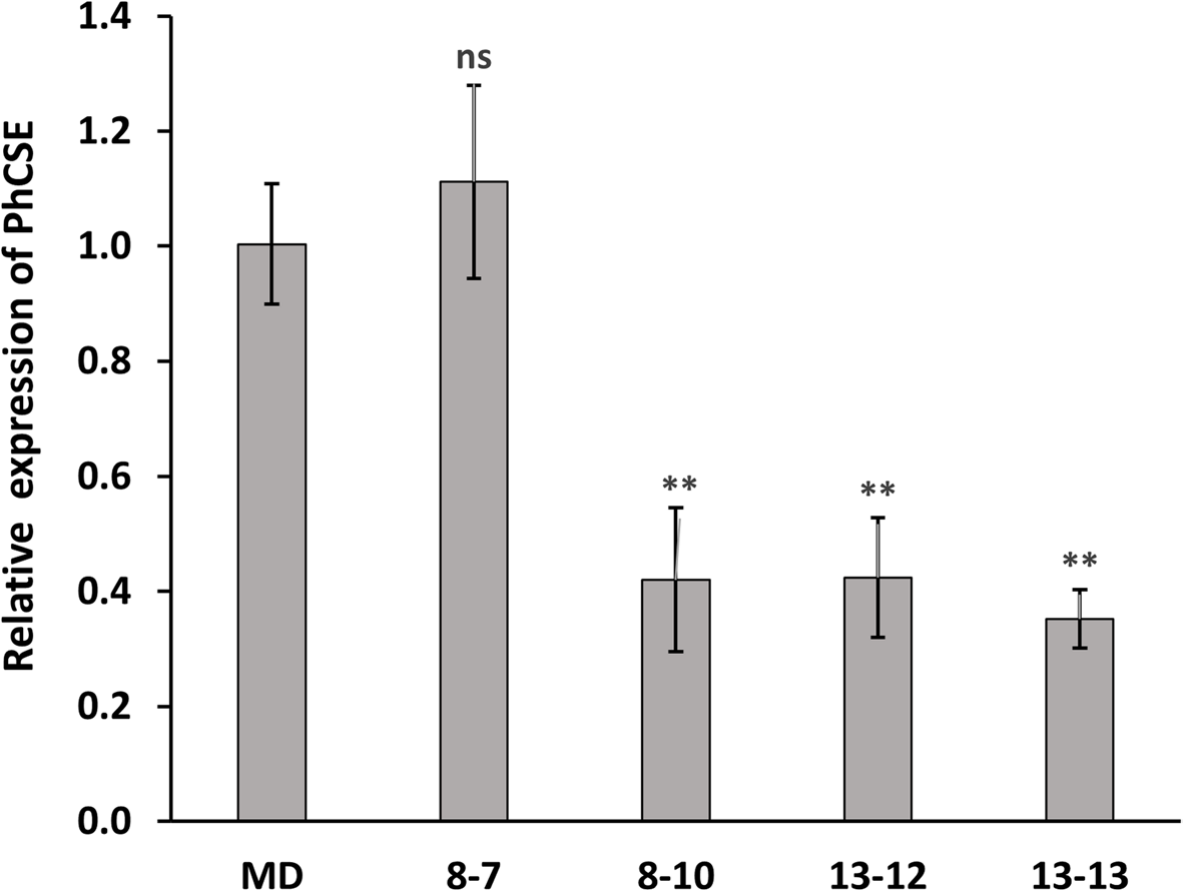
Gene expressions of *PhCSE* were quantified by the 2^−ΔΔCT^ method using quantitative RT-PCR (qRT-PCR) normalizing to the reference gene, *Ph18S r*RNA with three biological replications per *ir-PhCSE* plants (8–10, 13–12, and 13–13) and negative control line (8–7). Data show the relative transcription levels compared to *Petunia* x *hybrida* ‘Mitchell Diploid’ (MD) plants and the bars on the graphs indicate standard deviation. Asterisks (**) indicate that the transcriptions of *ir-PhCSE* plants are significantly different compared to MD (*p* < *0.001*, Student’s t-test; ns, not significant)

Through kanamycin selection and genomic DNA screening tests, three independent *ir-PhCSE* plants (8–10, 13–12, and 13–13) were finally selected from the T1 segregation lines. The endogenous transcript levels of *CSE* were reduced by more than 50% in three *ir-PhCSE* flowers than in MD with quantitative RT-PCR (qRT-PCR) analysis (Fig. 2). The transcript accumulation of line 8–7 was not different from MD but had gone through the transformation process, thus the 8–7 was considered as another reference to compare with *ir-PhCSE* plants. The phenotype of fully grown plants (three months old) was investigated, and *ir-PhCSE* plants were overall smaller than MD and 8–7 plants (Table 1). The *ir-PhCSE* plants were significantly shorter (11.31% ~ 30.87%), lower in the aerial fresh mass (23% ~ 55.8%) and had thinner stem diameters (20% ~ 23%) compared to MD plants. There was no significant difference in the number of branches, but all the *ir-PhCSE* plants had fewer branches than MD plants. The *ir-PhCSE* plants produced significantly more roots than MD plants, but the root lengths of the *ir-PhCSE* plants were not significantly different from MD plants. The flower phenotype varied among the *ir-PhCSE* plants (Table 2). Lines 8–10 and 13–13 had significantly thinner petioles, while line 13–12 had smaller stamens, than MD plants. Sepal lengths of lines 13–12 and 13–13 were significantly longer than MD plants.

**Table 1:** The phenotype analysis of *Petunia* x *hybrida* ‘Mitchell Diploid’ (MD) and *ir-PhCSE* T1 plants. Data represent mean ± SD (*n* = 3). The means were separated by one-way ANOVA and statistically significant differences (*p* < *0.05*) were identified by Tukey’s test. The results were shown by letters to represent differences among groups

Parameter	MD	8–7	8–10	13–12	13–13
Plant height (cm)	97.2 ± 2.3 a	93.5 ± 2.3 a	86.2 ± 3.8 b	82.5 ± 1.3 b	67.2 ± 2.3 b
Branch number ^z^	8.3 ± 1.5 a	7.7 ± 1.2 a	7.3 ± 2.3 a	5.3 ± 2.3 a	6.3 ± 2.3 a
Stem diameter (cm)	6.5 ± 0.6 a	5.5 ± 0.3 b	5.2 ± 0.4 b	5.0 ± 0.2 b	5.1 ± 0.1 b
Aerial fresh mass (g) ^y^	50.0 ± 9.1 ab	56.1 ± 6.4 a	47.7 ± 4.7 ab	22.1 ± 4.0 c	38.5 ± 4.7 bc
Root number ^x^	16.3 ± 2.5 c	25.0 ± 6.1 ab	32.3 ± 3.8 a	26.3 ± 5.0 ab	22.7 ± 2.1 bc
Root length (cm) ^x^	13.0 ± 1.4 a	15.2 ± 3.8 a	14.7 ± 4.2 a	13.1 ± 2.3 a	15.6 ± 4.7 a

^z^ Number of branches developed from the three thickest stems were counted

^y^Fresh mass of the three thickest stems was measured

^x^Root number and root length were determined from 4-week-old cuttings

**Table 2:** The phenotypes of reproductive parts were analyzed in *Petunia* x *hybrida* ‘Mitchell Diploid’ (MD) and *ir-PhCSE* T1 plants. Data represent mean ± SD (*n* = 3). The means were separated by one-way ANOVA and statistically significant differences (*p* < *0.05*) were identified by Tukey’s test. The results were shown by letters to represent differences among groups

Parameter	MD	8–7	8–10	13–12	13–13
Flower diameter (cm)	5.5 ± 0.2 a	5.3 ± 0.2 ab	5.6 ± 0.2 a	5.6 ± 0.1 a	5.2 ± 0.3 b
Petiole length (cm)	5.0 ± 0.2 a	4.9 ± 0.1 ab	4.7 ± 0.2 b	4.8 ± 0.1 ab	5.0 ± 0.1 a
Petiole diameter (mm)	1.8 ± 0.0 a	1.7 ± 0.1 ab	1.6 ± 0.1 b	1.7 ± 0.1 ab	1.6 ± 0.1 b
Sepal length (cm)	1.2 ± 0.1 c	1.7 ± 0.1 a	1.3 ± 0.0 c	1.5 ± 0.1 b	1.6 ± 0.1 b
Style length (cm)	4.5 ± 0.3 ab	4.1 ± 0.2 b	4.4 ± 0.7 ab	4.6 ± 0.1 ab	4.8 ± 0.1 a
Stamen length (cm)	4.5 ± 0.0 ab	4.5 ± 0.2 ab	4.5 ± 0.2 ab	4.4 ± 0.1 b	4.7 ± 0.1 a
Ovary length (cm)	0.5 ± 0.0 a	0.5 ± 0.0 a	0.5 ± 0.1 a	0.5 ± 0.0 a	0.5 ± 0.0 a
Anther head (mm)	1.5 ± 0.0 ab	1.7 ± 0.1 a	1.7 ± 0.2 a	1.3 ± 0.1 b	1.5 ± 0.1 ab
Stigma head (mm)	2.5 ± 0.1 bc	2.4 ± 0.1 c	2.7 ± 0.0 bc	2.7 ± 0.1 a	2.4 ± 0.1 c

### Volatile analyses of *ir-PhCSE* flowers

**Fig. 3:**
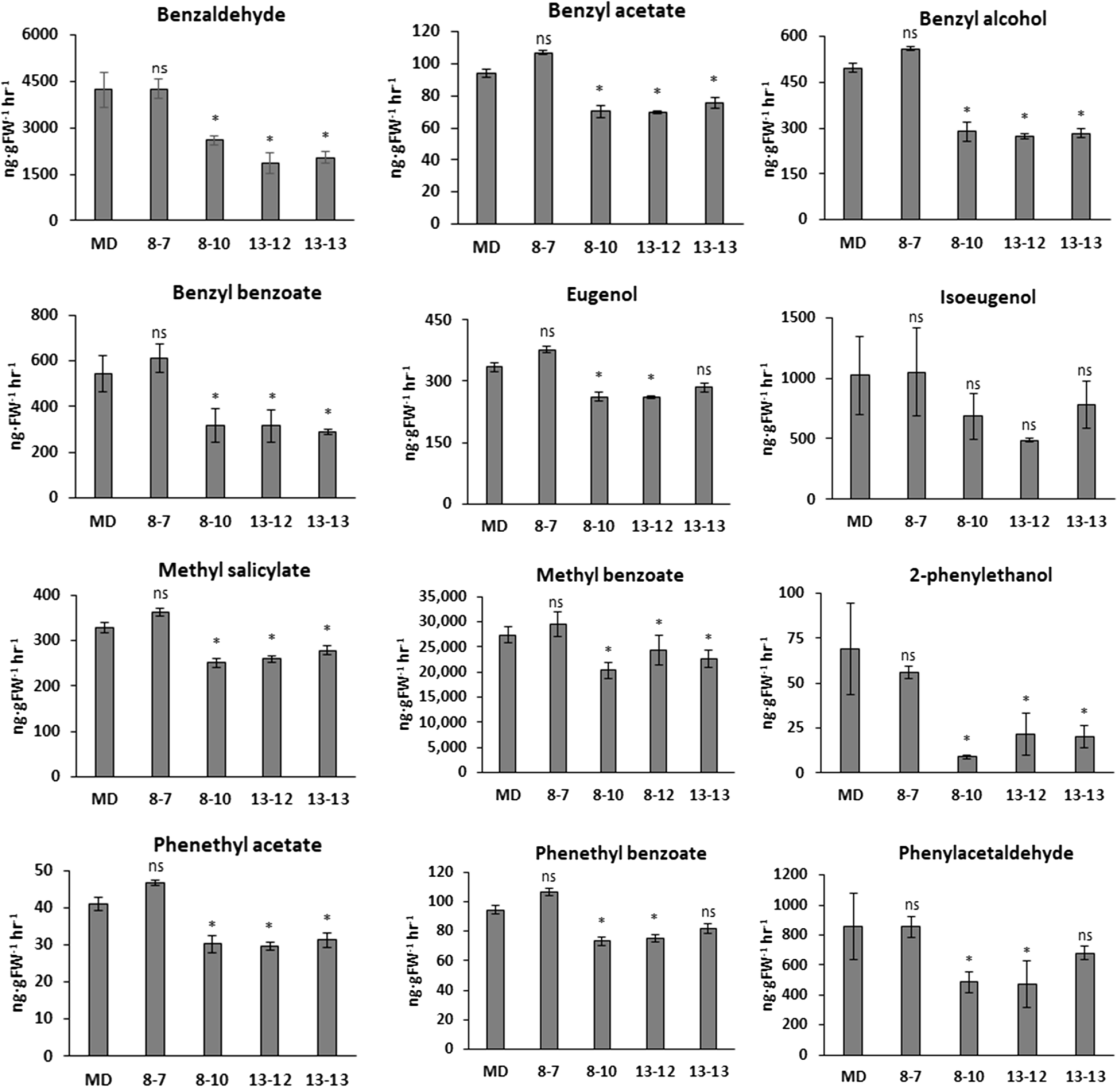
Floral volatile profiles of *Petunia* x *hybrida* ‘Mitchell Diploid’ (MD) and *ir-PhCSE* plants were identified by gas chromatography analysis. The fully opened flowers from three months old plants were harvested and the volatiles were collected for 1 h. The calculated emissions were compared to MD plants. Data represent mean ± SD, and the bars on the graphs indicate standard deviation. Asterisks (*) indicate that the volatile amounts of *ir-PhCSE* plants are significantly different compared to MD (*p* < *0.05*, Student’s t-test; ns, not significant)

**Fig. 4:**
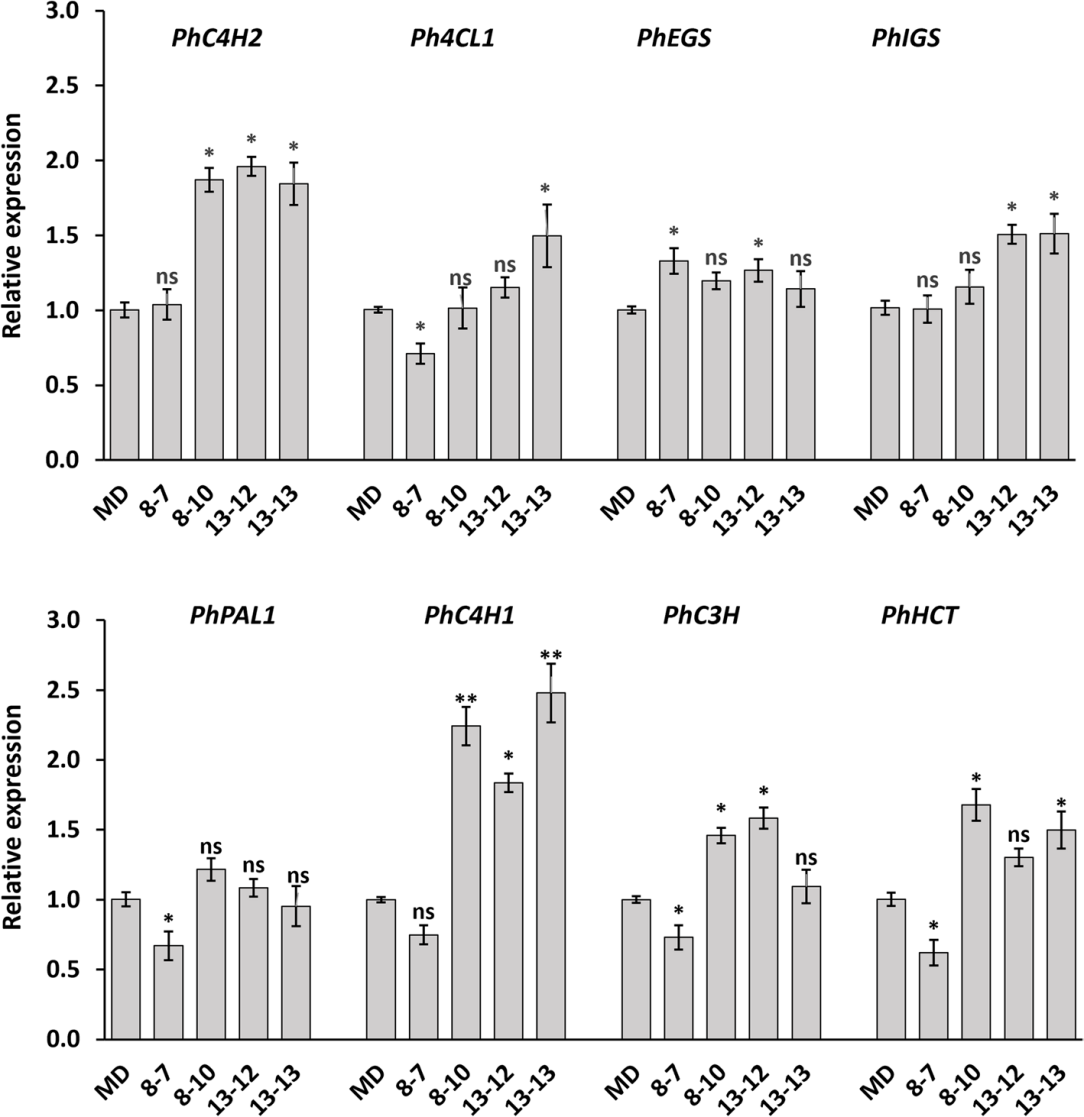
Eight FVBP gene expressions of petunia were quantified by the 2^−ΔΔCT^ method using quantitative RT-PCR (qRT-PCR) normalizing to the reference gene, *Ph18S r*RNA with three biological replications per *ir-PhCSE* plants (8–10, 13–12, and 13–13) and negative control line (8–7). Data represent mean ± SD. Data show the relative transcription levels compared to *Petunia* x *hybrida* ‘Mitchell Diploid’ (MD) plants and the bars on the graphs indicate standard deviation. Asterisks (*, **) indicate that the transcriptions of *ir-PhCSE* plants are significantly different compared to MD plants (*p* < *0.05, 0.001*, Student’s t-test; ns, not significant). *C4H*, cinnamate 4-hydroxylase; *4CL*, 4-coumaroyl: CoA ligase; *EGS*, eugenol synthase; *IGS*, isoeugenol synthase; *PAL*, phenylalanine ammonia lyase; *C3H*, *p*-coumarate 3-hydroxylase; *HCT*, hydroxycinnamoyl transferase

To evaluate the CSE function on FVBP, we analyzed floral volatiles of *ir-PhCSE* plants by GC–MS (Fig. 3). All three *ir-PhCSE* plants emitted significantly reduced amounts of benzaldehyde, benzyl acetate, benzyl alcohol, benzyl benzoate, 2-phenylethanol, and phenethyl acetate compared to MD flowers. Ten out of the twelve volatiles were lower in plants 8–10 and 13–12 than in MD plants, including eugenol, methyl salicylate, phenethyl benzoate, and phenylacetaldehyde. The amount of isoeugenol and methyl benzoate was also lower in *ir-PhCSE* plants, but not significantly different from MD plants. To investigate the relationship between FVBP genes and the volatiles, we did qRT-PCR (Fig. 4). Transcript levels of *PhPAL1* in *ir-PhCSE* plants were slightly different than MD plants, while transcripts of *PhC4H1* and *PhC4H2* in all three *ir-PhCSE* plants were more than double the transcripts in MD plants. The *ir-PhCSE* plants showed slightly elevated transcript levels of *PhC3H*, *PhHCT*, and *4CL* compared to MD plants, as well as of *PhIGS* and *PhEGS*.

### Analyses of lignin and phenolic compounds

**Fig. 5:**
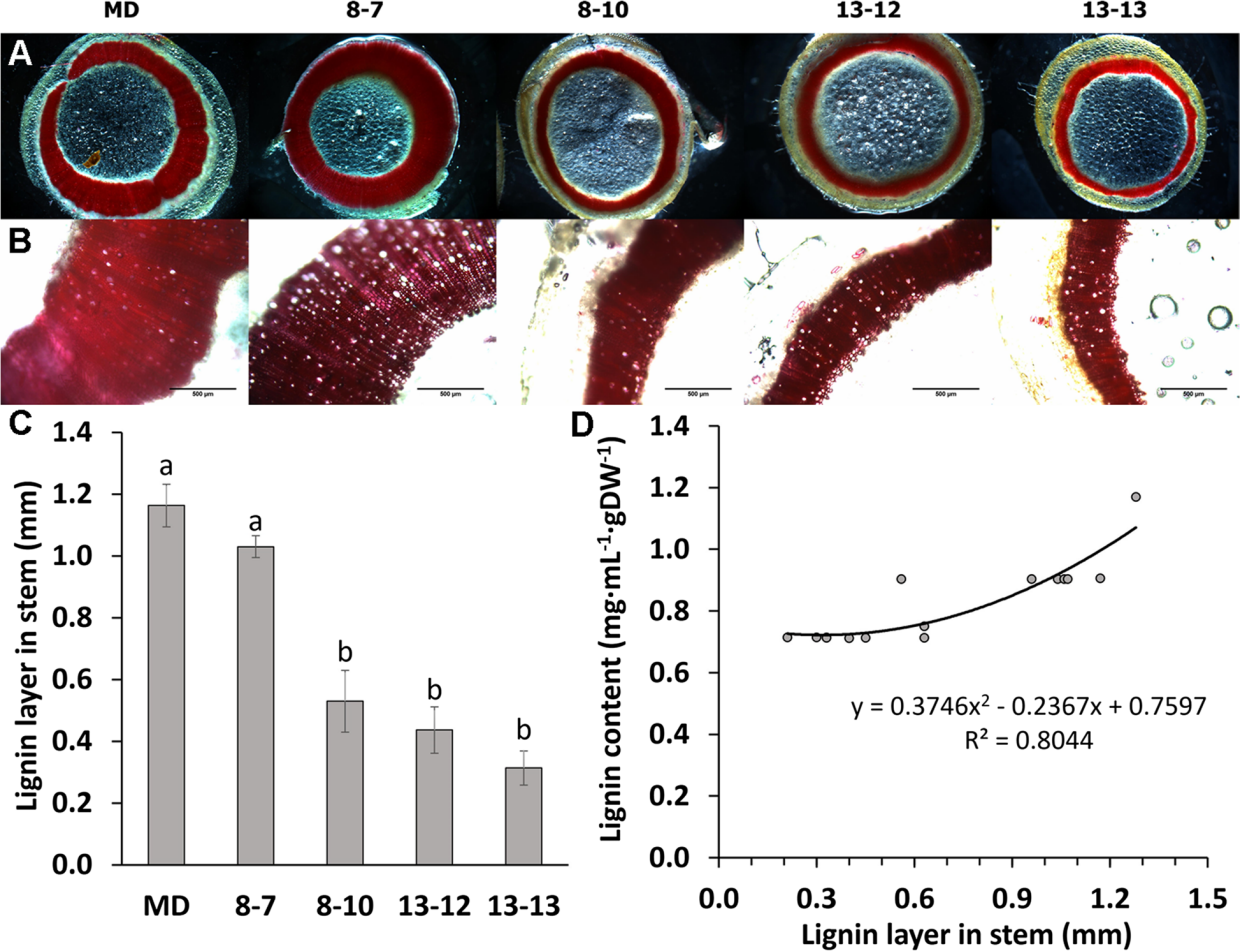
Lignin localized in stem was histologically analyzed. Stems of *Petunia* x *hybrida* ‘Mitchell Diploid’ (MD) and *ir-PhCSE* plants, were treated with 1% phloroglucinol with 50% HCl. Lignin was stained reddish in cross-sections of stems (**A**&**B**). Thickness of lignin layers of stem cross-sections were determined using image processing software, ImageJ (**C**). Regression test showed a significant positive correlation between lignin content in stem and thickness of lignin layer (*R*^*2*^ = *0.8044, P* < *0.01*) (**D**). Scale bars indicate 500 μm. Data represent mean ± SD. The means were separated by one-way ANOVA and statistically significant differences (*p* < *0.05*) were identified by Tukey’s test. The results were shown by letters to represent differences among groups

**Fig. 6:**
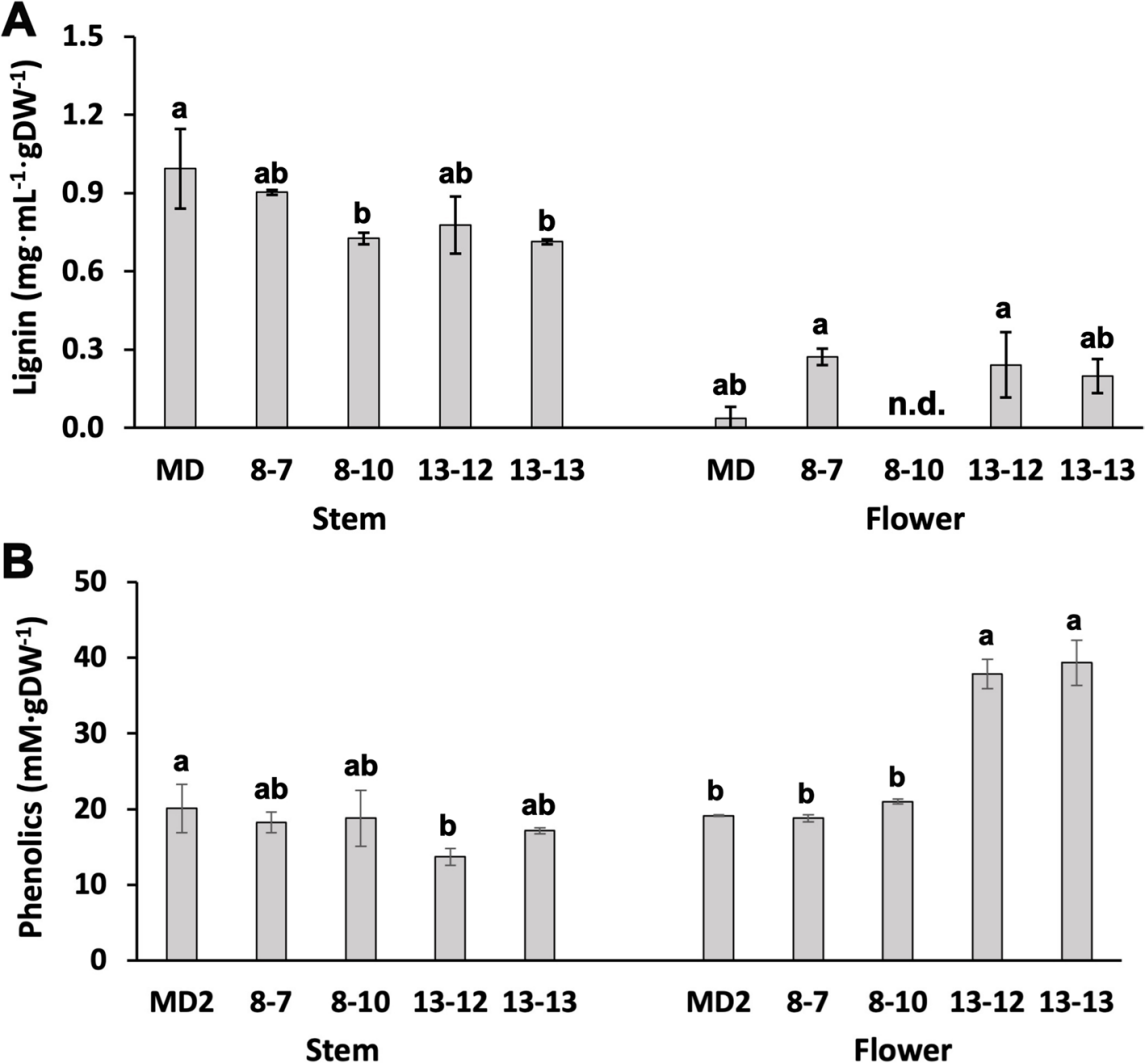
Lignin (**A**) and phenolic (**B**) compounds were quantitatively determined from stems and flowers of *Petunia* x *hybrida* ‘Mitchell Diploid’ (MD) and *ir-PhCSE* plants. Data represent mean ± SD. The means were separated by one-way ANOVA and statistically significant differences (*p* < *0.05*) were identified by Tukey’s test. The results were shown by letters to represent differences among groups and n.d. indicates not detected

When the stems were cross-sectioned and observed under a microscope, the lignin layers in the three *ir-PhCSE* plants were approximately 75% thinner than the lignin layers in the 8–7 and MD plants (Fig. 5B and C). In contrast, the stem diameters of the 8–7 and *ir-PhCSE* plants were not significantly different from each other, but all were significantly different from the stem diameter of the MD plants (Fig. 5A, B, and C and Table 1). There were no differences in the lignin layers of the flower tubes (Supplemental Fig. 1). The lignin amounts were significantly decreased in 8–10 and 13–13 stems, but the lignin amounts were not different in the *ir-PhCSE* flowers (Fig. 6A). A significant positive correlation (R^2^ = 0.8044) was found between lignin content and lignin layer thickness in the stems (Fig. 5D).

**Fig. 7:**
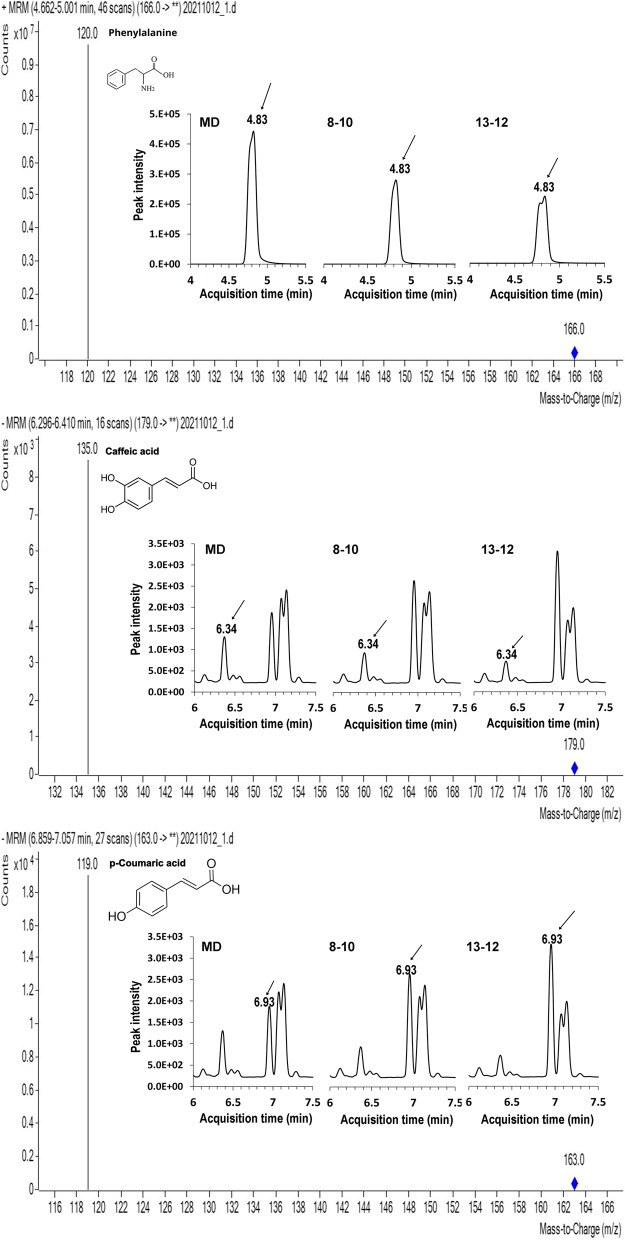
LC–MS chromatogram of primary MRM transition for phenolic compounds (phenylalanine, caffeic acid and *p*-coumaric acid) were determined from flowers of *Petunia* x *hybrida* ‘Mitchell Diploid’ (MD) and *ir-PhCSE* plants (8–10 and 13–12)

The amounts of phenolic compounds in 13–12 and 13–13 flowers were higher than in MD, but there were no significant differences in the total phenolic content in the stem (Fig. 6B). To further investigate the profile changes of phenolic compounds in the flowers, the phenolic extracts were measured by LC–MS (Fig. 7). The levels of phenylalanine and caffeic acid in *ir-PhCSE* flowers were significantly lower than in MD flowers. In *ir-PhCSE* flowers, quinic acid, shikimic acid, and trans-cinnamic acid decreased, while *p*-coumaric acid increased. The levels of caffeoyl quinate, ferulic acid, and coniferyl aldehyde in *ir-PhCSE* flowers were not significantly different from MD flowers (Table 3).

**Table 3:** Phenolic compounds were determined in *Petunia* x *hybrida* ‘Mitchell Diploid’ (MD) and *ir-PhCSE* T1 flowers with LC–MS (μg⋅mL^−1^⋅mgFW^−1^). Data represent mean ± SE (*n* = 3). The means were separated by one-way ANOVA and statistically significant differences (*p* < *0.05*) were identified by Tukey’s test. The results were shown by letters to represent differences among groups

Compound	MD	8–7	8–10	13–12	13–13
Quinic acid	4.7 ± 1.2 a	4.0 ± 0.9 a	4.0 ± 0.6 a	3.2 ± 0.8 a	4.2 ± 1.4 a
Shikimic acid	4.1 ± 1.3 a	4.2 ± 0.5 ab	2.7 ± 0.9 ab	N/A	2.0 ± 0.7 a
Phenylalanine	56. 7 ± 1.6 a	42.7 ± 0.8 a	34.4 ± 0.1 b	30.8 ± 1.0 b	36.1 ± 3.7 b
Caffeoyl quinate	0.1 ± 0.0 a	0.2 ± 0.1 a	0.1 ± 0.0 a	0.1 ± 0.0 a	0.2 ± 0.0 a
Caffeic acid	1.5 ± 0.1 a	1.6 ± 0.0 a	1.1 ± 0.0 b	0.7 ± 0.0 c	0.8 ± 0.1 bc
*p*-Coumaric acid	2.4 ± 0.7 a	2.3 ± 0.5 a	3.0 ± 0.2 a	3.3 ± 0.1 a	2.8 ± 0.6 a
*t*-Ferulic acid	8.2 ± 0.6 a	7.9 ± 0.4 a	8.3 ± 0.9 a	6.5 ± 0.2 a	8.0 ± 0.1 a
Coniferyl aldehyde	0.1 ± 0.0 a	0.1 ± 0.0 a	0.1 ± 0.0 a	0.1 ± 0.0 a	0.1 ± 0.0 a
*t*-Cinnamic acid	0.3 ± 0.0 a	0.2 ± 0.0 a	0.2 ± 0.0 a	0.2 ± 0.0 a	0.2 ± 0.0 a

## Discussion

### Reduced lignin content and altered growth in *ir-PhCSE* plants

According to Ha et al. [25], the class I CSE genes are involved in the reactions from caffeoyl-CoA to monolignols in dicots and monocots, although not in all species. Twelve out of the 61 species they investigated did not have the class I CSE genes, while a species of *Populus* had two CSE genes belonging to the class I CSE genes. Petunia *CSE* is homologous to the class 1 CSE genes of *Nicotiana sylvestris* (XP 009,777,258) and *Nicotiana tomentosiformisthe* (XP 009,593,524). The *CSE* down-regulated petunia plants had thinner lignin layers and lower lignin contents in the stems than MD plants. This result is consistent with other studies showing decreased lignin levels in CSE-modified plants [21–23, 25]. These results suggest that petunia CSE is essential for lignin synthesis, and it affects the carbon flux downstream of *p*-coumaric acid in petunia. However, changing the expression of *CSE* did not affect the lignin content in petunia flowers. The failure to find differences could be due in part to flowers having lower amounts of lignin overall, and the lack of an established lignin extraction method specifically for floral tissue. In this study, we applied the protocol for stem lignin extraction; however, the stem extraction protocol may not sensitive enough to detect variation in lower amounts of lignin.

The impacts of CSE on plant growth were varied. *CSE* mutants of Arabidopsis and *Medicago truncatula* showed dwarfism and altered developments [21, 23, 25], but *CSE* mutants of poplars did not show significant differences in plant growth [22, 24, 29]. *CSE* down-regulated petunia also showed stunted growth, so it is possible that the loss of CSE function inhibits growth and development more in vegetative plants. On the other hand, the lignin contents were lower in all mutants mentioned above, including poplar and Arabidopsis mutants. It shows the reduced lignin content has not resulted from the stunted growth and development of plants.

### Altered phenylpropanoid and volatile profiles in *ir-PhCSE* plants

According to Saleme et al. [22], the CSE gene could influence all the reactions from *p*-coumaroyl/caffeoyl/feruloyl/sinapoyl shikimate to the corresponding CoA thioesters. *CSE*-silenced poplars showed a different phenolic profile from the wild type, and the most abundant compound was *p*-coumaroyl/caffeoyl/feruloyl/sinapoyl shikimate. We analyzed FVBP gene transcripts and phenylpropanoids in *ir-PhCSE* flowers to investigate the effect of *PhCSE* on petunia volatiles. Increased *p*-coumaric acid content in *ir-PhCSE* flowers compared to MD flowers could be due to the alteration of *p*-coumaroyl/caffeoyl/feruloyl/sinapoyl acid synthesis pathways. Reduced *CSE* hinders the circulation of shikimate thus resulting in the accumulation of shikimates. Moreover, insufficient caffeic/ferulic/sinapic acids in *ir-PhCSE* flowers can limit the supply of their CoA thioesters, which are precursors of lignin polymers, thus leading to reductions of lignin in *ir-PhCSE* plants like in *CSE*-silenced poplar mutants [23].

Accumulated shikimates could make the high expression of *C3H*, *HCT*, and *4CL* compensate for the saturated shikimates. Elevated *p*-coumaric acid seems to lead to increased transcripts of *C3H* and *C4H*. A significant decrease in caffeic acid, but not in ferulic acid, in *ir-PhCSE* plants suggests that CSE is primarily involved in the reaction of caffeoyl shikimate to caffeic acid. The interaction between C3H and C4H with homo/hetero-dimer protein complexes is well known, and C3H is considered the main driver of protein–protein interactions in the endoplasmic reticulum (ER) [30, 31]. C3H/C4H complex has been shown to convert *p*-coumaric acid into caffeic acid [31], and HCT and 4CL are associated with C3H and C4H. The conversion of *p*-coumaroyl-CoA to *p*-coumaroyl shikimate by *HCT* and *4CL* increased the C3H and C4H complex [32].

The change in the transcript level of FVBP genes may be associated with C3H. In our previous study, we found down-regulation of *C3H* caused the severe reduction of most FVBP gene expression, including *C4H*, *HCT*, and *CSE* of *ir-PhC3H* plants [28]. C3H has been proposed as a regulator of petunia FVBP genes because most gene transcripts decreased in the *ir-PhC3H* plants. Because phenylpropanoid composition was altered in CSE-modified plants, it was expected that floral volatiles would be altered, too. The decreased phenylpropanoids of floral volatiles in the *ir-PhCSE* flowers suggests that CSE is associated with steps in the synthesis pathway of petunia FVBP. Like *CSE*-modified plants, *C3H* down-regulated plants showed dramatic alteration of many volatile compounds [28]. The volatiles in *ir-PhCSE* flowers might be directly affected by the expression of C3H because *ir-PhC3H* plants showed a much-altered profile of floral volatiles. We suggest that CSE affects the level of *C3H* transcripts and that the modified C3H control other petunia FVBP genes at a network level.

## Conclusions

We investigated *CSE* down-regulated petunias to investigate the roles of CSE on floral volatiles. Our results suggest that CSE plays a role in lignin composition and affects a carbon flux downstream of *p*-coumaric acid in petunia. In addition, the decreased transcripts of the CSE gene altered plant growth and volatiles of petunia. We suggest CSE works with C3H, HCT, and 4CL, influencing the petunia FVBP biosynthesis pathway downstream of *p*-coumaric acid.

## Methods

### Plant materials and growth measurements

*Petunia* x *hybrida* cv ‘Mitchell Diploid’ (MD) was a control for all experiments. The plants were grown in air-conditioned glass greenhouses in Florida. Plants were potted in PRO-MIX BX potting medium (Premier Tech Horticulture, PA) and fertilized with Scott’s Excel 15–5-15 (Scotts, Marysville, OH).

A 285 bp sequence of *PhCSE* (MF421742) amplified for RNAi vector construction (primers on Table S1). The RNAi was cloned in a pHK vector driven by a constitutive promoter, FMV and performed sanger sequencing at Genewiz from Azenta Life Sciences (South Plainfield, NJ). The vector was transformed into *Agrobacterium tumefaciens*, ABI. Five-week-old leaves after seed germination on ½ MS medium transformed according to the method of Jorgensen et al. [33]. All T0 and T1 tissues were collected for analyzing floral volatiles and transcript accumulation. Then the flowers were self-pollinated. T1 plants show 3:1 segregation by kanamycin screen and genomic DNA screen selected for further analyses. The University of Florida permitted the collection of plants and seeds and storage in the Plant Innovation Center of the University of Florida.

Growth characteristics of the *ir-PhCSE* plants were analyzed when fully grown, but root number and root length were measured using 4-week-old cuttings because fully grown plants had too many roots to count. The growth of the plants, including plant height, branch number, stem diameter, fresh mass, root number, and root length, were measured. The branch number and fresh weight were analyzed using the three thickest stems. Flower diameter, petiole length, petiole diameter, sepal length, style length, stamen length, ovary length, anther head diameter, and stigma head diameter were also measured using three representative flowers.

### Analysis of transcript accumulation

Total RNA was extracted using TriZOL™ (ThermoFisher Scientific, Waltham, MA) as previously described [34] and treated with TURBO™ DNA-free™ (Ambion Inc., Austin, TX). 50 ng µL^−1^ of RNA was prepared after measuring the concentration using a NanoDrop™ 2000c spectrophotometer (ThermoFisher Scientific, Waltham, MA). Transcript accumulation was analyzed by semi-quantitative (sq)RT-PCR using a One-step RT-PCR kit (Qiagen Co., Valencia, CA) and quantitative (q)RT-PCR using Power SYBR® Green RNA-to-CT™ 1-Step kit and StepOnePlus™ real-time PCR system (ThermoFisher Scientific, Waltham, MA). Primers were designed using Primer3 (https://primer3.ut.ee/) (primers on Table S1). To analyze transcript accumulation of different FVBP genes in *ir-PhCSE* plants, (q)RT-PCR was performed using *PhFBP1* as an internal standard with three replications to compare the expression of each gene. All (q)RT-PCR data were analyzed using 2^−∆ΔCt^ method [35].

### Analysis of floral volatiles

Fully opened flowers were harvested at 18.0 h, and volatiles was collected for 1 h in glass tubes using a push–pull dynamic headspace collection system as previously described [28, 36]. Volatiles were collected from three biologically replicated flowers on glass columns containing approximately 50 mg HaySep Q 80–100 porous polymer adsorbent (Hayes Separations Inc., Bandera, TX) and eluted with methylene chloride. Quantification of volatiles in the elution matrix was performed on an Agilent 7890A Series gas chromatograph (GC) equipped with an Agilent 5977A single quadrupole mass spectrum detector (MSD) using an equipped DB-5 column (Agilent Technologies, Santa Clara, CA). The volatile mass emission rates (ng*gFW^−1^*hr^−1^) were calculated based on each compound’s individual peak area relative to the peak area of an elution standard, nonyl acetate, within each sample and standardized for each sample’s corresponding biological mass. Mean separation and comparison of *ir-PhCSE* floral volatiles to MD controls was performed with Tukey’s multiple range test (one-way ANOVA, P < 0.05) using a JMP Pro v.12 statistical software package (SAS Institute Inc., Cary, NC).

### Histochemical staining analysis

To find the localization of lignin in petunia tissues, hand-cut transverse sections from the bottom 3 cm of stems and flower limb tubes made for lignin staining [37]. Stems were incubated in 70% ethanol containing 1% phloroglucinol overnight, and the limb tubes were for 1 h. After rinsing the samples with water, a few drops of 50% HCl were added to the sections. The stem sections observed under a fluorescence stereo microscope (MZ16F, Leica Microsystems, Wetzlar, Germany) and the relative intensities of the fluorescent images were determined using ImageJ software (1.51; National Institutes of Health, Bethesda, MD) as described by Cho et al. [38].

### Analyses of phenolic compounds and lignin contents

Phenolic compounds and lignin were extracted according to the method of Stadnik and Buchenauer [39]. Stems 20 cm from the bottom of each plant were dried at 80 ֯C for 18—24 h and then ground. 0.5 g of the powder was soaked in 4 mL of 50% methanol at 80 ֯C for 1.5 h. After centrifugation, the supernatant (free phenolic compounds) was used for the Folin–Ciocalteau assay. The remained tissue was treated with 2 mL of 0.5 M NaOH at room temperature for 24 h. Then, 0.5 mL of 2 M HCl was added and centrifuged. The supernatant (wall-bound phenolics) was used for the Folin–Ciocalteau assay. The remained tissue was used for lignin assay. For phenolic compounds analysis, 950 uL of water was added to 50 uL of the supernatant followed by 50 uL of 2 M Folin–Ciocalteau (Millipore Sigma, St. Louis, MO) and 100 uL of 20% Na_2_CO_3_. After 20 min, the absorbance of the samples was measured at 725 nm, and the content was calculated using a standard curve of *p*-coumaric acid (Millipore Sigma, St. Louis, MO).

For the lignin assay, 50 mg of dried tissue was incubated in 5 mL of thioglycolic acid in 2 M HCl (1:10, v/v) at 100 ֯C for 4 h, cooled on ice, and centrifuged at 3000 g for 5 min. The pellet was washed twice with 2 mL of water and incubated in 5 mL of 0.5 M NaOH at 4 ֯C for 18 h. After centrifugation, 200 uL of HCl was added to the supernatant and incubated on ice for 4 h. After centrifugation, the pellet was washed twice with 0.1 M HCl and then resuspended in 0.5 N NaOH. The absorbance of the samples was measured at 280 nm, and the content was calculated with a standard curve of lignin (Millipore Sigma, St. Louis, MO).

### LC–MS analysis of phenolic compounds

Phenolic compounds were extracted from fully opened flowers. 100 mg of ground tissue was incubated in 1 mL of methanol at 70 ֯C for 30 min with shaking, followed by centrifugation for 10 min. The supernatant was dried all, and the pellet was dissolved in 150 uL of methanol and transferred after centrifuging for 10 min.

Phenolic compounds were analyzed using reversed-phase high-performance liquid chromatography and electrospray ionization tandem mass spectrometry (LC–ESI–MS/MS). An Agilent 1260 HPLC system (Agilent Technologies, Santa Clara, CA) connected to an Agilent 6430 Triple Quadrupole MS system was used. Separations were performed at 35 °C using an InfinityLab Poroshell 120 EC-C18 column (3.0 × 150 mm, 2.7 µm) with a flow rate of 0.5 mL/min. The mobile phase consisted of water with 0.1% formic acid (solvent A) and acetonitrile with 0.1% formic acid (solvent B), and the column was eluted with a gradient of solution B over time: 0 min with 2.5%, 2 min with 5%, 6 min with 45%, 13 min with 50%, 13.1 to 14 min with 2.5%. The total run time was 14 min, and the column equilibration time was 6 min. The triple quadrupole MS system was equipped with an ESI source operating in negative and positive ionization mode with the following parameters: source gas temperature, 300 °C; drying gas (nitrogen) flow, 10 L/min; nebulizer pressure, 30 psi; capillary voltage, 4000 V. The data were acquired and processed by Agilent MassHunter LC/MS Data Acquisition software (version B.06.00). Flow injection analysis of single-standard solutions was used (Supplemental Table 2). The limits of detection (LOD) and quantitation (LOQ) for phenolic acids were established at a signal-to-noise ratio (S:N) of 3:1 and 10:1, respectively. The data were analyzed using MassHunter Qualitative (B.06.00) and Quantitative (B.06.00) software (Agilent Technologies, USA).

## Supplementary Information

**Additional file 1: Supplemental Figure 1.** Lignin localized in flowers was histologically analyzed. The flowers of *Petunia *x* hybrida *‘Mitchell Diploid’ (MD) and *ir-PhCSE* plants were treated with 1% phloroglucinol with 50% HCl. Lignin was stained reddish in cross-sections of flower tubes. **Supplemental Table 1.** Primer sequences used in this research. **Supplemental Table 2.** MRM transition of single standards.

## Data Availability

The datasets generated and/or analysed during the current study are available in the NCBI repository, https://www.ncbi.nlm.nih.gov/bioproject/935294.
