# Supplementary material for: Altered profile of floral volatiles and lignin content by down-regulation of Caffeoyl Shikimate Esterase in Petunia
Source: BMC Plant Biol. 2023 Apr 22;23:210. doi: 10.1186/s12870-023-04203-0 (PMC10122356; doi:10.1186/s12870-023-04203-0)
Supplement: Supplementary file 1 — Additional file 1: Supplemental Figure 1. Lignin localized in flowers was histologically analyzed. The flowers of Petunia x hybrida ‘Mitchell Diploid’ (MD) and ir-PhCSE plants were treated with 1% phloroglucinol with 50% HCl. Lignin was stained reddish in cross-sections of flower tubes. Supplemental Table 1. Primer sequences used in this research. Supplemental Table 2. MRM transition of single standards. [file 12870_2023_4203_MOESM1_ESM.docx]

**Supplemental figure and Tables**

Supplemental Figure 1. Lignin localized in flowers was histologically analyzed. The flowers of *Petunia* x *hybrida* ‘Mitchell Diploid’ (MD) and *ir-PhCSE* plants were treated with 1% phloroglucinol with 50% HCl. Lignin was stained reddish in cross-sections of flower tubes.

Supplemental Table 1. Primer sequences used in this research.

Supplemental Table 2. MRM transition of single standards.


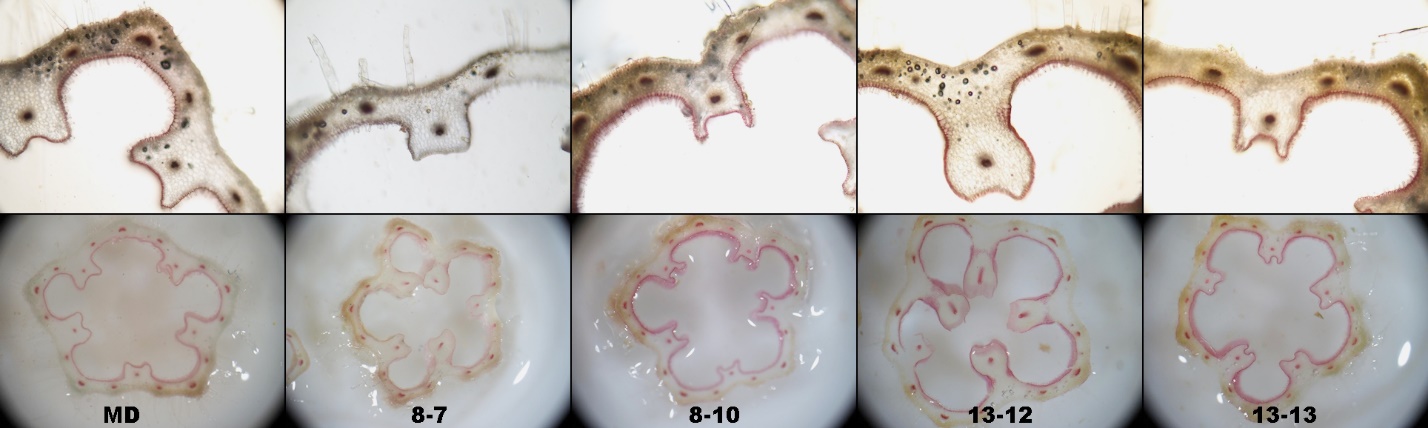


Supplemental Figure 1. Lignin localized in flowers was histologically analyzed. The flowers of *Petunia* x *hybrida* ‘Mitchell Diploid’ (MD) and *ir-PhCSE* plants were treated with 1% phloroglucinol with 50% HCl. Lignin was stained reddish in cross-sections of flower tubes.

| Supplemental Table 1. Primer sequences used in this research. | | |
| --- | --- | --- |
| Name | Forward | Reverse |
| PhCSE-RNAi | 5’-GGGGTTATGCTGTGTTTGCTGC-3’ | 5’-CCTTGGATGGCTTCATTAGCTC-3’ |
|  |  | 5’-CGCTCAACCCTCTCATCAATC-3’ |
| PhCSE- qPCR | 5'-GGTGTCCACCATTCCAAATCC-3' | 5'-GCCAACCTGTGTCAGAACCAT-3' |
| Ph4CL-qPCR | 5’-GGCCTGTGCTGGCTATGTGT-3’ | 5’-AGCTGCCGGGTCATTTAGGT -3’ |
| PhC3H-qPCR | 5’-TTTGGGAAGAGGTTTGTGAA-3’ | 5’-GTCAAGGGGGAACATCCA -3’ |
| PhC4H1-qPCR | 5’-AGCAGGTGTAACAAACTGCAA-3’ | 5’-AAACTGGGACAGGGATAGGA-3’ |
| PhC4H2-qPCR | 5’-AACTTGTCCAAACAAAAATGGA-3’ | 5’-TGGCAATTTAAAACGTTTGCT-3’ |
| PhEGS-qPCR | 5’-TGCTGTGGAGCCAGCTAAGT-3’ | 5’-GTGGCTCCTGGCTGAACCAA -3’ |
| PhIGS-qPCR | 5'-AGGAAGATGTCGCAGCCTACA-3' | 5'-GTTTGCTGCCCTTGGATCAT-3' |
| PhHCT-qPCR | 5'-GACTATGGTGAAGCCAGCAA-3' | 5'-ACATAGCCTCCCAGCCATAG-3' |
| PhPAL1-qCPR | 5’-GCTAGGCGGTGAGACGCTAA-3’ | 5’-CTCGGACAGCTGCACTGTCA-3’ |
| Ph18S-qPCR | 5'-TTAGCAGGCTGAGGTCTCGT-3' | 5'-AGCGGATGTTGCTTTTAGGA-3' |
| PhFBP1-qPCR | 5’-TGCGCCAACTTGAGATAGCA-3’ | 5’-TGCGCCAACTTGAGATAGCA-3’ |
| PhUbiq-qPCR | 5’-GTCGATGGTCTTTGTTAGTGTTGTGT-3’ | 5’-GTCGATGGTCTTTGTTAGTGTTGTGT-3’ |

| Supplemental Table 2. MRM transition of single standards. | | | | | | |
| --- | --- | --- | --- | --- | --- | --- |
| Compound | Precursor Ion (m/z) | Product Ion (m/z) | Fragmentor Voltage (V) | Collision Energy (V) | Cell Accelerator Voltage | Mode |
| (-)-Quince acid | 191 | 85 | 135 | 33 | 3 | Negative |
| Shikimic acid | 173 | 93 | 135 | 18 | 3 | Negative |
| Phenylalanine | 166 | 120 | 65 | 15 | 4 | Positive |
| Chlorogenic acid | 353 | 191 | 90 | 12 | 1 | Negative |
| Caffeic acid | 179 | 135 | 90 | 14 | 1 | Negative |
| *p*-Coumaric acid | 163 | 119 | 90 | 12 | 1 | Negative |
| *t*-Ferulic acid | 192.9 | 133.9 | 105 | 17 | 1 | Negative |
| Coniferyl aldehyde | 177 | 162 | 90 | 12 | 2 | Negative |
| *t*-Cinnamic acid | 146.9 | 102.9 | 95 | 13 | 1 | Negative |
